# Draft genome sequence of *Enterobacter sp*. from wheat grain

**DOI:** 10.1128/mra.00576-23

**Published:** 2024-01-18

**Authors:** Niradha Withana Gamage, Tehreem Ashfaq, Tiffany Chin, Janice Bamforth, Sean Walkowiak

**Affiliations:** 1 Grain Research Laboratory, Canadian Grain Commission, Winnipeg, Canada; University of Maryland School of Medicine, Maryland, USA

**Keywords:** wheat, bacteria

## Abstract

Here, we report the draft genome sequence of an isolate from the *Enterobacter cloacae* species complex. *Enterobacter* spp. are plant growth-promoting microbes and biocontrol agents. Analyses of this genome will serve as a useful resource for future studies of similar microbes isolated from grain.

## ANNOUNCEMENT

*Enterobacter cloacae* is a facultative anaerobic, gram-negative rod, which inhabits different environments including soil, water, plants, and animals. Individuals within the *Enterobacter cloacae* species complex have been beneficial in the agricultural sector due to their plant growth-promoting microbe and biocontrol activity. They are associated with nitrogen fixation, soil phosphorous solubilization, and enhancement of soil porosity. They also have potential to suppress soil-borne pathogens by production of siderophores, and various antimicrobial compounds such as bacteriocins, chitinases, and antibiotic resistance proteins (1, 2).

We announce the draft genome of an isolate of the *Enterobacter cloacae* species complex recovered from Canadian Western Red Spring wheat kernels obtained in Canada in 2018. The organism was isolated from a grain sample soaked in Tryptic Soy Broth medium and incubated at 37°C for 24 h. The culture was subsequently streaked onto Chromagar solid medium (BioRad Inc.) once and incubated at 37°C for 24 h to obtain pure colonies. Genomic DNA was extracted from a single colony using QIAgen Blood and Tissue kit (QIAgen Inc., Canada). Sequencing was performed by Illumina HiSeq using a 2 × 150 paired-end protocol, generating 4,298,983 reads with sequence coverage of 240×. Read quality was assessed using FastQC v. 0.11.9.

Assembly was performed using shovill v.1.1.0, and assembly summary statistics were generated with QUAST v.5.1.0 (3). The assembly produced 103 contigs over 1,000 base-pairs (bp) in length, with an *N*50 value of 434,283 bp and an *L*50 of 4. The genome size was 4,823,888 bp, and average GC content was 55.47%. Genome annotation and gene predictions were performed using Prokka v.1.14.6 (4), which identified 4,877 coding sequences, 89 tRNA genes, and 15 rRNA genes. The isolate was identified as being part of the *Enterobacter cloacae* species complex using the PubMLST database and default parameters of mlst v.2.23.0 for multilocus sequence typing, as well as by Refseq_masher matches.

Furthermore, functional annotation of the genes by Prokka revealed that this isolate may harbor multiple genes associated with nitrogen metabolism (*nasA*, *nasD*, *nasR*, and *nirD*), phosphorus solubilization (*phoB*, *phoE*, *phoH*, *phoP*, *phoQ*, *phoR*, *phoU*, and *gcd*), siderophore production (*FhuA*, *FhuB*, *FhuC*, *FhuD*, *FhuE*, *FhuF*, *FepA*, *FepB*, *FepC*, *FepD*, *FepE*, *FepG*, and *EntS*), iron transport regulation (*EfeB*, *EfeU*, *EfeO*, *FeoA*, *FeoB*, and *FeoC*), genes for flagella function (*flgA*, *flgB*, *flgC*, *flgD*, *flgE*, *flgF*, *flgG*, *flgH*, *flgI*, *flgJ*, *flgK*, *flgL*, *flgM*, *flgN*, *flhA*, *flhB*, *flhC*, *flhD*, *flhE*, *fliA*, *fliD*, *fliE*, *fliF*, *fliG*, *fliH*, *fliI*, *fliJ*, *fliK*, *fliM*, *fliN*, *fliO*, *fliP*, *fliQ*, *fliR*, fliS*, fliT*, *fliY*, and *fliZ*), and chemotaxis (*CheA*, *CheB*, *CheR*, , *CheV*, *CheW*, *CheY*, and *CheZ*) that could be beneficial for plant growth promotion and/or defense (5–10).

## Data Availability

The annotated draft genome sequence data have been deposited to NCBI, under the GenBank accession number JASIUE000000000.1 and RefSeq accession number GCF_030369015.1. The BioProject database accession number of the organism is PRJNA973081, and the Sequence Read Archive information for this project is available under the accession number SRR24650830.
